# Experiences of supervised high-intensity interval training—a motivator for exercise maintenance among patients with rheumatoid arthritis: a qualitative interview study

**DOI:** 10.1136/bmjopen-2025-106750

**Published:** 2025-12-25

**Authors:** Gunilla Limbäck, Annelie Bilberg

**Affiliations:** 1 Department of Orthopaedics, Institute of Clinical Sciences, Sahlgrenska Academy, University of Gothenburg, Gothenburg, Sweden; 2 Department of Occupational Therapy and Physiotherapy, Sahlgrenska University Hospital, Gothenburg, Sweden; 3 Department of Health and Rehabilitation, Physiotherapy, Institute of Neuroscience and Physiology, Sahlgrenska Academy, University of Gothenburg, Gothenburg, Sweden

**Keywords:** Physical Therapy Modalities, RHEUMATOLOGY, QUALITATIVE RESEARCH, Exercise

## Abstract

**Abstract:**

**Objective:**

High-intensity interval training (HIIT) has been shown to improve cardiovascular health and physical fitness in patients with rheumatoid arthritis (RA). However, patient perspectives on this type of exercise remain insufficiently explored. The aim of this study was to describe experiences of supervised HIIT among patients with RA and to elucidate how patients maintain exercise on their own after completing the supervised intervention.

**Design:**

A qualitative study based on semistructured in-depth individual interviews analysed using qualitative content analysis.

**Participants:**

Patients were recruited from a multicentre randomised controlled trial in Western Sweden. Twenty patients (16 women) with RA who had participated in the high-intensity exercise programme for 12 weeks were included in this qualitative interview study.

**Setting:**

The supervised HIIT was conducted in a hospital setting.

**Results:**

The results were divided into two domains, corresponding to the two broad open-ended questions. The first domain comprises four categories: (1) challenging and effective with HIIT, (2) belonging to a group facilitates exercise, (3) feeling confident when exercising and (4) stimulating to feel the effect of exercise on the body. The second domain comprises two themes: (1) taking responsibility for one’s health and (2) a feeling of resignation.

**Conclusion:**

HITT was perceived as challenging yet manageable. Follow-up visits with a knowledgeable physiotherapist were requested, since exercise was perceived as a lifelong treatment and just as crucial as pharmacological treatment. Most patients described taking responsibility for their health and maintaining their exercise regime after the supervised intervention; however, some expressed a feeling of resignation and were unready for lifestyle changes. Nevertheless, having RA can be a motivator when exercise is viewed as an important part of medical treatment.

**STRENGTHS AND LIMITATIONS OF THIS STUDY**

- To strengthen the study’s credibility, patients from four separate exercise groups from two different hospitals participated in the interviews.
- The heterogeneity in age, sex and ethnicity among the patients interviewed is considered a strength.
- The patients had low-to-moderate disease activity, which may limit the generalisability of these findings to patients with higher disease activity.

## Introduction

Rheumatoid arthritis (RA) is a chronic inflammatory disease that causes systemic inflammation and peripheral arthritis,^1 2^ often leading to deterioration in physical function^3^ and general health.^4^ Aside from anti-rheumatic pharmacological therapies,^5 6^ physical activity (PA), structured exercise^7^ and physiotherapy^8^ are recommended for optimal RA management. Patients with RA have an increased risk of comorbidities,^9^ mainly cardiovascular diseases (CVDs).^10^ Physical inactivity and a low cardiorespiratory fitness (CRF) level are associated with increased CVD risk,^11^ while exercise, especially aerobic exercise, has been shown to have positive effects on CRF and reduce the CVD risk in RA.^12^ However, only a minority of patients with RA engage in exercise at a high enough level to improve CRF.^11^

Patients with RA report several barriers to PA and exercise in general,^13 14^ some of which are arthritis-specific, such as pain and fatigue.^15^ Additionally, a lack of clarity from healthcare about appropriate PA and exercise regimes in RA has been found to hamper health-improving PA and exercise.^16^ Moreover, studies have reported low levels of exercise maintenance following a period of structured exercise in patients with RA.^17 18^ The reasons behind this decline have only been minimally explored.^13 19^

High-intensity interval training (HIIT) is an exercise mode involving bouts of high intensity at a heart rate maximum (HRmax) of 90%–95%, interspersed with periods of lower intensity.^20^ HIIT has previously been shown to have beneficial effects on CRF in healthy people^21^ and patients with inflammatory joint diseases.^22^,^24^ A recent multicentre randomised controlled trial by our study group revealed that supervised exercise combining HIIT with strength exercise improved cardiovascular health, physical fitness and overall health without increasing pain or disease activity in patients with RA.^25^ A qualitative study reported that patients with axial spondyloarthritis (SpA) perceived HIIT as a challenging but manageable type of exercise.^26^ Since patients with RA and axSpA may face different challenges related to exercise, an exploration of the perspectives of patients with RA on supervised HIIT and maintaining exercise on their own can provide useful in-depth understanding of their experiences. The aim of this study was to describe experiences of supervised HIIT among patients with RA and elucidate how patients maintained exercise on their own after completing the supervised exercise intervention.

## Patients and methods

### Study design

A qualitative research design based on individual semi-structured interviews was chosen for data collection, and content analysis with an inductive approach was conducted to analyse the texts.^27^ The consolidated criteria for reporting qualitative research guided the reporting of the study.^28^ We used the Standards for Reporting Qualitative Research (SRQR)^29^ to draft this manuscript, and the SRQR reporting checklist when editing is provided in online supplemental A.

### Patients

Patients with RA were recruited from a multicentre randomised controlled trial in Western Sweden.^25^ The inclusion criteria were as follows: patients allocated to the intervention group with high intensity exercise who had completed at least 50% of the supervised sessions. In addition, all patients fulfilled the criteria for RA according to the 1987 American College of Rheumatology or the 2010 European Alliance of Associations for Rheumatology,^2 30^ with a disease duration >1 year, an age of 20–60 years, stable treatment on antirheumatic drugs for the past 3 months, low-to-moderate disease activity and the ability to speak and understand Swedish. Twenty patients from four different exercise groups were purposefully selected to ensure diversity in sex, age, study site and exercise group. An information letter about the interview study was sent to the patients, who were subsequently contacted by phone. All the contacted patients agreed to participate in the interviews. Each respondent signed an informed consent form before the start of the interview. The study protocol and consent documents were approved by the National Ethics Committee in Sweden (No. 2022-03511-02).

### Exercise programme

The exercise protocol has been previously described in detail.^25^ In brief, the exercise was individually tailored based on physical capacity and ability and was supervised two times per week for 12 weeks by a physiotherapist in a hospital setting. The HIIT session included high-intensity bouts (90%–95% HRmax) interspersed with periods of lower intensity (70% HRmax). The strength exercise included large muscle groups, 2–3 sets, with 8–10 repetitions, at 70%–80% of one repetition maximum (1RM). A third non-supervised aerobic session (70% HRmax) of the patient’s choice was encouraged. The non-supervised exercise was determined through dialogue between the patient and the physiotherapist to facilitate regular health-improving PA over time.

Different motivation strategies were applied during the exercise intervention, including exercise guidance following the principles of self-efficacy and motivational support, the identification of possible limitations and barriers, an individual exercise diary, the use of heart rate monitors and continuous dialogue with individual feedback by the physiotherapist. Four physiotherapists who were clinical experts within rheumatology supervised the exercise.

### Patient and public involvement

A patient research partner from the Swedish Rheumatism Association was involved in the study design and choice of open-ended questions to the interview guide.

### Data collection

Data were collected through interviews. An interview guide with open-ended questions was developed, and answers to two broad, open-ended questions were analysed: *how did you experience the supervised high-intensity interval exercise? How did you experience continuing to exercise after the supervised exercise programme ended?* The interview guide is presented in online supplemental eFile B. The interviews took place between October 2022 and October 2023, 3–6 months after the HIIT sessions ended, at Sahlgrenska University Hospital, at Uddevalla Hospital or at a location of the patient’s choice via a digital meeting with video. A pilot interview was first conducted to assess the effectiveness of the interview format. No revisions were made, and the pilot interview was included in the analyses. The interviews lasted 35–105 min, with a median of 55.5 min. Inclusion was continued until data saturation was reached, whereby no new information addressing the research questions was given, and existing data were being replicated. The interviews were conducted by the first author (GL) who did not assist in the intervention, a female physiotherapist with extensive experience in the rehabilitation of patients with various musculoskeletal problems. The second author (AB) is also a female physiotherapist with long experience within rheumatology and rehabilitation of patients with RA. Both authors hold PhD degrees and have experience in qualitative research.

### Data analysis

The interview texts were analysed by content analysis.^27^ The audio-recorded interviews were transcribed verbatim. The interviews were then read multiple times to grasp their content. Thereafter, the text was condensed into meaning units and abstracted into codes. The analytical process moved back and forth between the whole and parts of the text. The answers to the two broad, open-ended questions led to two different analyses that were linked yet separated. The first question resulted in four categories and 10 subcategories. In contrast, the second question generated richer material allowing a deeper, more latent and interpretative analysis that resulted in the development of two themes and their related subthemes.^31^ To strengthen the trustworthiness of this process, the two authors engaged in an open and critical dialogue, discussing all steps of the analysis until consensus was reached.

## Results

A total of 20 patients (16 women) participated in the interviews after completing the supervised intervention, both native Swedes and immigrants were represented (table 1). The results are divided into two domains (tables2  3).

**Table 1:** Characteristics for the patients with rheumatoid arthritis participating in the interviews

Characteristics	
Sex female/male, n	16/4
Age, years, median (range)	54.0 (33-59)
Disease duration, year, median (range)	3.0 (1-25)
Disease activity	
DAS28-ESR, median (range)	1.8 (0.1–2.9)
Pharmacological treatments	
Conventional DMARD n (%)	16 (80)
Biological DMARD n (%)	11 (55)
Targeted synthetic DMARD n (%)	2 (10)
Corticosteroids n (%)	0 (0)
Supervised exercise sessions, n, median (range)	20 (13–24)
Non-supervised sessions, n, median (range)	14 (4–21)

DAS28-ESR, Disease Activity Score in 28 joints using the Erythrocyte Sedimentation Rate; DMARD, disease-modifying antirheumatic drug.

**Table 2:** Results of the first question: patients’ experiences of supervised HIIT

Categories	Subcategories
1. Challenging and effective with HIIT	HIIT: demanding yet manageable
	Fast, fun and effective to perform HIIT
	Motivating to endure tough exercising with RA
2. Belonging to a group facilitates exercise	Committing to the group
	Community with others having RA
3. Feeling confident when exercising	Individual guidance from a specialist physiotherapist
	Increased knowledge of exercise
4. Stimulating to feel the effect of exercise in the body	Perceived physiological effects
	Reduced RA symptoms
	Mental well-being from exercise

HIIT, high-intensity interval training; RA, rheumatoid arthritis.

**Table 3:** Results of the second question: patients’ experience of maintaining exercise after the intervention

Themes	Sub-themes
1. Taking responsibility for one’s own health	Exercising as part of daily life
	Improved confidence to exercise
	Conditions for maintaining exercise
	Exercising as part of medical treatment
2. A feeling of resignation	Needing extended guidance and support for exercise
	Not having the will to prioritise health
	Being disappointed over unfulfilled expectations

### Description of the patients

Before the intervention, approximately half of the patients described participating in exercise, while a few patients had minimal or no exercise experiences. Two patients reported having a previous ankle fusion surgery and one a neck fusion surgery; therefore, they needed the exercise to be further adapted to account for their physical limitations.

### Results of the first question: patients’ experiences of supervised high-intensity interval training

A majority of the patients described the supervised HIIT as a positive experience. The patients’ experiences were grouped into four categories: 1) *challenging and effective with HIIT*, 2) *belonging to a group facilitates exercise*, 3) *feeling confident when exercising* and 4) *stimulating to feel the effect of exercise in the body*. Each category further included two or three subcategories, as outlined in table 2;, the ID number after the quotations refers to individual patients.

#### Challenging and effective with HIIT

This category was grouped into three subcategories.

##### High-intensity interval training: demanding yet manageable

The high-intensity intervals were described as extremely demanding and tough to perform, as the patients had never pushed themselves this hard before. However, the shortness of the bouts of high intensity and the interspersed periods of lower intensity were described as facilitating the exercise and making the strenuous exercise more manageable.

*It’s like short intervals, where you push yourself a bit and then you get to rest, and so on, and you could notice results quite quickly. The first time you were supposed to cycle, and you knew you wouldn’t go so high, but then you were supposed to go higher. Then you thought, oh no, I’m going to die; this will never work.* (ID 1)

The participants also described focusing on biking and monitoring their heart rate as beneficial. Still, a few patients who were not used to exercising found the sweating and elevated heart rate uncomfortable and expressed a need for reassurance from the physiotherapist that the exercise was being performed correctly.

##### Fast, fun and effective to perform high-intensity interval training

The patients expressed that the HIIT was both fast-paced and enjoyable to perform. Some commented that attending the HIIT sessions felt effortless and was something they did not want to miss. They described the sessions as seeming to pass more quickly and to be more effective in comparison with traditional, continuous aerobic exercise. The HIIT provided them with a new dimension of training, and they were pleased to acquire new skills.

*If you can manage to maintain the intervals, it’s much better, and I’m really happy with this type of workout – or whatever you’d call it. I haven’t done HIIT before. It was completely new to me. Of course, I’ve heard about it, but I’ve never embraced it. It has given me a new dimension on how to exercise*. (ID 18)

##### Motivating to endure tough exercising with rheumatoid arthritis

The patients expressed that they initially believed that it would be impossible to exercise at such a high intensity with an RA diagnosis. Some patients said that before the study, they were uncertain how to exercise with a rheumatic disease and believed that exercise would lead to increased joint pain. However, these fears were not fulfilled; on the contrary, the patients managed to engage in much tougher exercise than they had thought possible, and this new and positive experience made them motivated to continue exercising at a higher intensity.

*I didn’t really think you could push yourself so hard, but it was all good – I felt much more energetic in my body and felt both physically and mentally much better too, in my opinion*. (ID12)

### Belonging to a group facilitates exercise.

This category was grouped into two subcategories.

#### Committing to the group

Patients expressed a sense of responsibility to participate in the supervised exercise sessions, as they believed that their presence contributed to the group dynamic.

*A bit of a motivator (with the group) because it’s easier to back out if you’re alone, you don’t have anyone to consider*. (ID 10)

The small, closed group heightened their sense of accountability, creating a permissive and supportive atmosphere that made the exercise easier to manage.

*I liked that we were a group, so it wasn’t just me and a leader, but I also liked that it was a small group. It always motivates you to exercise with someone, and even if we didn’t know each other, you create your little exercise relationship. On those tough mornings, it motivates you to come. Yes, but I’m there, I contribute to the team and I contribute with my energy and exercise*. (ID 20)

#### Community with others with RA

The patients expressed a sense of affinity with the other patients with RA and found mutual understanding for each other’s challenges. Some patients commented that this community helped them feel less lonely in dealing with their diagnosis. Exercising with others—some of whom were even worse off than themselves—was described as a motivator for exercise, particularly for those who were newly diagnosed.

*Above all, you can see that they have the same problems, they also have pain, and it can be tough because I’m still in that phase where I’m often angry that I can’t do certain things. When my hands don’t work properly and it hurts, and I have pain in my feet, pain in my knees, I get so irritated, but here, everyone had the same issues, like, ‘no, I can’t do certain strength exercise because it hurts there’, and there were those who had it much worse than I do, I think*. (ID 15)

### Feeling confident when exercising

This category was grouped into two subcategories.

#### Individual guidance from a specialist physiotherapist

Patients emphasised the importance of the support from a specialised rheumatology physiotherapist who understood their individual challenges and could individually tailor the exercise to meet their specific needs.

*You know that they* (the physiotherapists) *have that knowledge with them, so it’s definitely a sense of security because then you dare to invest in a different way than if you expose yourself to something you haven’t done before and have someone with you who might not know either, so it was definitely a sense of security, they had their expertise, and they could teach*. (ID 16)

Simultaneously, they were challenged with new and more demanding exercises. They described a sense of safety and reassurance when guided by a knowledgeable physiotherapist, which empowered them to commit to the exercise.

*It was nice to get some tips as well, and in the exercises, you were also challenged, or I was challenged*. (ID 20)

#### Increased knowledge of exercise

Patients described gaining valuable knowledge about exercise. They learnt how to identify an appropriate load level during the HIIT session by using a heart rate monitor, which made exercising more manageable and effective. They also learnt how to exercise and adjust their exercise based on their current RA symptoms. Additionally, they felt well equipped with tools that boosted their confidence to continue exercising.

*I have realised what kind of exercise is necessary to feel good, so it has created health benefits for me*. (ID 2)

### Stimulating to feel the effect of exercise in the body.

This category was grouped into three subcategories.

#### Perceiving physiological effects

Patients expressed feeling stronger, fitter and happier. They experienced increased energy and a positive sensation in their bodies.

*That I feel strong, that I could endure more and not just in terms of stamina, but it was a combination of being able to handle all the exercising, manage work and still have energy left, and it was a very nice feeling, so I like that feeling*. (ID 3)

They also noted that it became easier to perform various daily activities, such as climbing stairs and opening jars. The improvements in their overall physique contributed to a greater sense of independence. Moreover, exercising led to additional health benefits such as weight loss and decreased waist circumference; some patients also experienced easier management of their diabetes.

*I felt that since I live on the third floor without an elevator and carrying things, I have hesitated to buy too much if it’s heavy, but I noticed that I became stronger, and then I could wash my car, like, yes, and I could do it myself, so I really noticed a difference, and I became, well, it was just positive, simply*. (ID 6)

#### Reduced rheumatoid arthritis symptoms

Patients described reduced joint problems, including decreased pain and increased mobility.

*I rarely had flare-ups, but now my condition has reached a higher level. A more stable level, so there has been an improvement in the upper tier, if you know what I mean.* (ID 2)

A reduced sense of fatigue and a need for fewer pain relievers were highlighted as resulting from the exercise. Some also experienced a reduction in relapses of the disease; one patient even reported complete cessation of antirheumatic medication after the intervention period.

*I have felt so much better, allowing me to refrain from taking my medication. And thinking about the health aspect, that I don’t have to go to my doctor… and I noticed that I felt better and better and waited to take the medication. I never took it again.* (ID 18)

#### Mental well-being from exercise

Patients described an increased sense of well-being after exercise, which made it easier to manage their pain and other symptoms. They found that their reduced discomfort created a positive mindset. Several patients reported improved sleep quality after exercising, which in turn increased their stamina. Moreover, they felt a greater sense of mental ease and experienced better concentration.

*I think you become a bit more positive and get a different mindset in some way. I don’t think about it as much, that I have pain, like I did before.* (ID 19)

*But one thing I didn’t expect, which was very positive, was that I became much calmer because of the exercise, so even if a lot was happening at work or if something became stressful, I could handle it better and more calmly.* (ID 4)

### Results of the second question: patients’ experiences of maintaining exercise after the intervention

The majority of the patients expressed that they had participated to a high degree in the supervised exercise, but the patients’ ability to maintain the exercise on their own varied. A total of 15 patients said that they had continued to exercise after the supervised exercise period had ended, whereas five patients did not. The patients’ experiences with maintaining their exercise were grouped into two themes: (1) *taking responsibility for one’s health* and (2) *a feeling of resignation* (table 3). The ID numbers after the quotations refer to individual patients.

#### Taking responsibility for one’s health

This theme was grouped into four sub-themes.

##### Exercising as part of daily life

Some patients said that they continued with the same exercise regimen after the supervised exercise, while others adjusted the exercise based on their newly achieved knowledge, noting that they had received tools that made it possible to maintain exercise on their own. Still, some patients expressed frustration that the supervised exercise stopped at the end of a semester, at a time when routines were likely to be broken, and that the responsibility for continuing to exercise fell entirely on themselves.

*In the end, I can find it quite boring, but I see the benefit of exercising, like it can be worth the boring parts*. (ID 9)

Patients described how the exercise had become a natural part of their lives, as they wanted to stay healthy and continue to live a good life. They did not perceive any barriers to continuing with their exercise routine, and they organised their daily lives around their exercise sessions, planning for lifelong exercise. Some also developed meaningful activities in terms of exercising with their partner, which further integrated the exercise into their daily lives.

*These days, my partner and I work out together, and then we go grocery shopping afterward. Now we plan our daily routine around the exercising. It’s really nice doing something together.* (ID 1)

##### Improved confidence to exercise

The knowledge the patients gained from the physiotherapist, along with their experiences from the supervised exercise, boosted their understanding of how to adapt exercise to their personal conditions and rheumatic disease, both overall and regarding symptom fluctuations. This increased their self-confidence to exercise. Many patients reported continuing their exercise routines with confidence, believing it was a well-suited way to manage their RA. Based on the new knowledge they gained during the study and on their increased confidence, some patients described overcoming anxiety about going to a gym on their own.

*It’s about daring and really pushing, daring to push and see what happens with your body. You think so much about what you can handle. No, but I stop here at this weight, or I push this much because I might be in pain tomorrow. Sure, you might be in pain tomorrow, but it’s not that pain, it’s muscle soreness instead*. (ID 1)

##### Conditions for maintaining exercise

Feeling the effect of exercise in their bodies was described as a key factor that motivated the patients to maintain their exercise. Many patients expressed the importance of having an exercising companion for sustained exercise over time. Moreover, setting personal goals was expressed as facilitating exercise. Easy access to training facilities, a gym and outdoor running trails was also highlighted as facilitators. The ability to use an individual heart rate monitor during exercise further encouraged them, as it allowed them to track their heart rate and optimise their performance. Flexible working hours and a high degree of autonomy at work were also described as facilitating exercise habits.

*I think accessibility is important. That way, I go straight from work to the gym before getting in the car and heading home because otherwise a lot of the energy and motivation disappears before you even get home.* (ID 10)

*It’s important to change clothes and put on workout gear, then I know I’ll put in more effort. Unlike if I exercise in regular clothes, for example. For a power walk session, it’s not just like going out for a lunch walk, because those are two different walks for me.* (ID 19)

##### Exercising as part of medical treatment

Patients expressed that they view exercise as a form of medicine, equally essential as pharmacological treatment for optimal management of their rheumatic disease.

*It might motivate me a bit to exercise as well, knowing that I don’t want to walk around with canes or barely be able to move or function at home. I can use the medications available. I can also exercise when I know it makes me feel good, that’s what I can influence.* (ID 10)

They emphasised that, just like medication, exercise must be performed on a regular basis to have an effect, which made them continue with exercise to prevent deterioration of their RA. In addition, they pointed out that exercise should be modified and followed up in the same way as medical treatment; thus, they requested regular follow-ups with a physiotherapist for modification of their exercise.

### A feeling of resignation

This theme was grouped into three subthemes.

#### Needing extended guidance and support for exercise

Some patients expressed that they needed continuing supervision from a physiotherapist for guidance and motivation, as they felt unable to manage exercise on their own. Some even expressed a desire for lifelong supervised exercise at the hospital. A lack of previous exercise experiences and a feeling of not having enough knowledge of the RA disease were described as hindrances to exercising on their own.

The participants emphasised the importance of exercising in a group with commitment, support and scheduled times, which they considered would have enabled them to continue exercising. This point had also been noted during the intervention period, where one session per week was designated for self-directed exercise—a task some patients found challenging to perform on their own.

*I can’t do it alone, I can if I’m in a group or if I have someone leading. Say now we take a few more steps, and now, it’s just this song left. I don’t have that in myself to push myself on, no, I don’t have that, no*. (ID 7)

#### Not having the will to prioritise health

Some patients said that while they could intellectually understand that exercise was good for their health, they lacked the will and energy to incorporate it into their daily lives. Patients who stopped exercising directly after the end of the supervised period expressed a sense of resignation, as they now faced the too-high barrier of starting all over again.

*I had forgotten to exercise or, well, I just ignored it. I think I decided that it’s no use now that I haven’t kept it up.* (ID 6)

Even greater resignation was expressed by those who said that they did not know what could motivate them to maintain their exercise, as they realised that they now prioritised other things over exercise. Stress related to work, such as long working hours and a heavy workload, was described as a barrier to exercise. Moreover, concerns about exercising in a gym, including not enjoying the mentality of gyms and the cost of a gym, were expressed as barriers to maintaining exercise.

*There are other things that are more important to me in life than exercise.* (ID 11)

#### Being disappointed over unfulfilled expectations

Patients commented that even though they had never succeeded in sustaining exercise in the past, they had had high expectations for starting and maintaining exercise when they signed up for the study. While they were able to adhere to the supervised exercise, they found it difficult to maintain exercise on their own. They described how previous feelings of fear of failure and feelings of insecurity when comparing themselves with others more accustomed to exercising returned after the supervised exercise, when they were supposed to exercise on their own. Moreover, the patients had hoped to experience the ‘rush’ from exercise described by others, and the disappointment of not feeling it made it harder for them to continue exercising.

*I actually thought after this HIIT exercising that I would feel more energetic, and get that boost, but I didn’t feel that…. it has never happened for me, unfortunately.* (ID 7)

A few patients also described how negative experiences, such as unpleasant memories from childhood gymnastic classes or a history of compulsive behaviour related to exercise and diet, still negatively impacted their attitudes towards exercise; these were expressed as a barrier to continued exercise.

## Discussion

This study shows that patients with RA can complete demanding HIIT exercise while receiving professional guidance and support from a physiotherapist. The patients expressed that it was stimulating to feel the effects of exercise, and most patients took responsibility for their health and maintained the exercise on their own after the end of the intervention period; however, some expressed a feeling of resignation and were unready for lifestyle changes.

Some patients described how, before participating in the study, they were concerned about exercising at high intensity with an RA diagnosis and had negative expectations of increased joint pain due to exercise. This perception aligns with previous studies reporting that the RA disease itself can be a barrier to exercising^14 17^ and that patients unaccustomed to exercise express doubts about the benefits of exercise and concern that exercise will increase their pain.^32^ In the present study, the patients overcame their negative expectations after attending the supervised exercise intervention; instead, they experienced increased physical ability and overall higher mental well-being, which made it easier to manage their pain and other symptoms. At the same time, they experienced fewer relapses of the disease and less joint pain; some even reduced their intake of antirheumatic medications (disease-modifying antirheumatic drug). These results indicate that the guidance from the physiotherapist based on the principles of self-efficacy and motivational support, together with individual modifications of the exercise based on their symptoms and flare-ups, strengthened patients’ ability to exercise at a high-intensity level. According to Bandura,^33^ individuals are more motivated to follow guidance given by people they trust and feel connected to.

The patients experienced HIIT as challenging and demanding, yet manageable and enjoyable, as they felt the positive effects of the exercise on their body. They expressed that it was motivating to endure tough exercising despite their RA diagnosis, which aligns with a study of HIIT for patients with axSpA.^26^ A major difference between the two patient groups is that patients with RA tend to be less active at a vigorous intensity level than patients with axSpA. The findings from the present study and previous results from our study group^25^ demonstrate that patients with RA can tolerate high-intensity exercise without risking a deterioration in disease activity or increased pain, indicating that supervised high-intensity exercise could be a recommended treatment for patients with RA.

In this study, patients who managed to continue exercising described positive physical and mental effects during the supervised exercise and felt that they could implement their new knowledge and skills in their own exercise regimes after the supervised intervention. Some of these patients had previous experiences with exercise, although not with HIIT, and said that, once they got started, they had managed to maintain this new exercise routine.

Motivation and PA behaviour change can be understood and explained using the self-determination theory (SDT).^34^,^36^ In SDT, different types of motivation are described along a continuum. At one end of the continuum is intrinsic motivation, the most self-determined or autonomous form of motivation, when a person performs the behaviour voluntarily because it feels inherently interesting or enjoyable. At the other end of the continuum is amotivation, which represents a lack of any intention or motivation to engage in the behaviour. Extrinsic motivation is found between the two ends of the continuum.^37^ It can represent a highly self-determined regulator style, where a person engages in a behaviour to attain specific outcomes associated with that behaviour.^38^

From an SDT perspective, some patients in this study shifted toward more extrinsic forms of motivation, as they changed their behaviour and experienced a sense of achievement related to their exercise. The positive experiences during the intervention may have enhanced their self-efficacy, which in turn motivated them to take greater responsibility for their health by maintaining exercise. A previous study showed that patients with arthritis who believe in the positive benefits of exercise and possess higher self-efficacy feel more confident in their ability to exercise, regardless of disease severity.^39^ This is consistent with the findings of the present study, in which some patients regarded exercise as a vital component of their treatment. They described feeling more motivated to maintain their exercise routines to prevent deterioration of their RA. Additionally, they expressed that the exercise became enjoyable and fun, and they began planning for lifelong PA. These experiences suggest a shift toward a more autonomous form of motivation, which is associated with sustained behaviour change. This interpretation is further supported by findings from another study indicating that strenuous exercise is associated with intrinsic motivation.^40^

In contrast to those who continued exercising, some patients did not manage to independently maintain their exercise after the intervention concluded. They described feelings of resignation towards exercising and not having the will to prioritise their health or implement lifestyle change. Studies have shown that frustration^41^ and amotivation^42 43^ are predictors of exercise dropout. These patients also described having to overcome a high threshold to be able to attend training facilities or gyms and expressed the need for an extended period of support to manage the transition to exercising on their own. Some described negative experiences of exercising from childhood and school gymnastics that still affected their attitude towards exercise, indicating low self-efficacy and amotivation for exercising. Interestingly, patients who appeared amotivated to exercise on their own were able to adhere to the supervised exercise. This may indicate extrinsic motivation, particularly as they expressed a desire to continue having access to supervised exercise.

Most patients requested follow-up visits with a physiotherapist after the supervised exercise ended to update their exercises and help with motivational support, regardless of whether they were used to exercising or not before the study began. Moreover, the patients expressed an awareness that exercise is an adjunct treatment to pharmacological treatment for managing RA; therefore, they considered follow-up visits with a physiotherapist as equivalent to follow-up visits with a physician for medical treatment.

Support from family, friends and colleagues was stressed as important for maintaining exercise. This was expressed both by patients who had support and succeeded in maintaining exercise and by those who lacked support and did not manage to maintain exercise. A supportive environment, positive social influences and constructive social changes are known to facilitate maintenance of behaviour change.^14 44 45^

In this study, two signs were identified during the supervised intervention and linked to failure to maintain exercise: difficulties performing the non-supervised sessions once a week during the intervention period and not having previous experiences with exercise. These findings align with the results of o a study showing that past exercise behaviour predicts future behaviour.^46^ According to a previous study,^47^ people have different and often motivationally conflicting reasons for engaging in exercise, which makes analyses of motivation within the field of exercise behaviour challenging.

### Clinical implications

HIIT is an exercise intervention that can be implemented for patients with RA, since it is effective and well tolerated. It is important for patients to be initially offered individual guidance from a physiotherapist specialised in rheumatology to tailor the exercise to each person’s symptoms and physical capacity. We believe that a small, closed group with other people with similar rheumatic diseases increases patients’ commitment to exercise, which is in line with the results of other studies.^26 48 49^ Follow-up visits after a supervised exercise period can stimulate patients to maintain their exercising. Moreover, patients with no exercise experiences and those who cannot manage to complete exercise sessions on their own during the supervised period should be identified and given extra motivational support.

### Strengths and Limitations

A qualitative research design is appropriate for this type of study, and qualitative content analysis is a commonly used analysis method in healthcare. Trustworthiness was ensured throughout the process, from the planning of the study to the analysis and implications. The two researchers are experienced with qualitative research and aware of the importance of taking a critical view of oneself and one’s research methods.^50^ Moreover, the researchers have different clinical backgrounds, with one from rheumatology and the other from orthopaedics, which helped to broaden their perspectives. However, there might have been a slight risk that the researchers missed information in the interviews and the analysis owing to their pre-understanding of the phenomenon.^50^ To strengthen the study’s credibility, patients from four separate exercise groups from two different hospitals (one in a smaller town and one in a bigger city) were invited to participate in the interviews. Additionally, the heterogeneity in age, sex and ethnicity of the study population enriched the material and is regarded as a strength of this study. The patients had low-to-moderate disease activity; the generalisability of the findings to patients with a higher disease activity may be limited. However, low disease activity has become increasingly common among patients with RA owing to the advances made in pharmacological treatment.^51^

To address the study’s dependability, the two researchers collaborated on the data analysis,^52^ which yielded results in two separate domains. The first question resulted in categories and subcategories. For the second question, the material was richer and led to a deeper analysis with a more latent and interpretative content, resulting in two themes. The different levels of analysis might be seen as a weakness,^46^ although the two domains of the analysis are clearly separated, which we consider a strength rather than a weakness.

Qualitative research has limited generalisability; however, we consider transferability to be relevant when planning supervised high-intensity exercise for patients with well-controlled RA and when considering different approaches to support patients in maintaining exercise.

### Future research

The motivational process to support exercise maintenance after a period of supervised HIIT among patients with RA requires further investigation. Moreover, health economic evaluations of the continued provision of supervised HIIT for patients unable to maintain exercising on their own require further investigation.

## Conclusion

In this work, patients with RA perceived HIIT as challenging yet manageable and found the intervention to be effective. Individual guidance from a physiotherapist specialised in rheumatology gave the patients confidence to perform HIIT. Follow-up visits with a knowledgeable physiotherapist were requested, since exercise was perceived as a lifelong treatment and just as crucial as pharmacological treatment.

Most patients described taking responsibility for their health and maintaining their exercise regime after the supervised intervention, but some expressed a feeling of resignation and were not ready for lifestyle changes. The motivational process is complex, and maintaining exercise among patients with a chronic disease such as RA is even more complex. Nevertheless, having RA can be a motivator when exercise is viewed as an important part of medical treatment.

## Supplementary material

10.1136/bmjopen-2025-106750 online supplemental file 1

10.1136/bmjopen-2025-106750 online supplemental file 2

## Data Availability

Data are available upon reasonable request.
