# Supplementary material for: Experiences of supervised high-intensity interval training—a motivator for exercise maintenance among patients with rheumatoid arthritis: a qualitative interview study
Source: BMJ Open. 2025 Dec 25;15(12):e106750. doi: 10.1136/bmjopen-2025-106750 (PMC12742093; doi:10.1136/bmjopen-2025-106750)
Supplement: online supplemental file 2 [file bmjopen-15-12-s002.pdf]

Supplement B

Interview guide

1. How did you experience the supervised high-intensity interval exercise?
   Follow-up questions:
   How did you experience exercising in a group?
   How did you experience the supervised exercising?
   How did you experience exercising on your own?
   Did you have any problems with your body during the exercising?
   If so, how did you handle the problems?

2. How did you experience continuing to exercise after the supervised exercise programme ended?
   Follow-up questions:
   Did you continue to exercise after the supervised exercising?
   What has made it easier to continue exercising?
   What has made it difficult or prevented you from continuing exercising?
   How did you overcome the obstacles?
   How have you been able to motivate yourself to continue exercising?
   What support would you have needed to facilitate continued exercising?

To conclude, is there anything more you´d like to share?
