# Supplementary material for: Experiences of supervised high-intensity interval training—a motivator for exercise maintenance among patients with rheumatoid arthritis: a qualitative interview study
Source: BMJ Open. 2025 Dec 25;15(12):e106750. doi: 10.1136/bmjopen-2025-106750 (PMC12742093; doi:10.1136/bmjopen-2025-106750)
Supplement: Supplementary file 3 [file bmjopen-2025-106750.reviewer_comments.pdf]

PEER REVIEW HISTORY

BMJ Open publishes all reviews undertaken for accepted manuscripts. Reviewers are asked to complete a checklist review form and are provided with free text boxes to elaborate on their assessment. These free text comments are reproduced below.

ARTICLE DETAILS

Title (Provisional)

Supervised high-intensity interval training – a motivator for exercise maintenance among patients with rheumatoid arthritis. A qualitative interview study

Authors

Limbäck, Gunilla; Bilberg, Annelie

VERSION 1 - REVIEW

**Reviewer**	**1**
**Name**	**Kennedy, Norelee**
**Affiliation**	**University of Limerick, Clinical Therapies**
**Date**	**15-Aug-2025**
**COI**	**None**

This is an interesting topic of increasing interest to those working in and researching in rheumatology and to people with RA.

There are however a number of aspects of the manuscript that need further consideration and/or revision - see attached file for further details.

There is increasing interest in HIIT for rheumatoid arthritis, however, there is still little evidence on it as a treatment and on patient views and experiences of HIIT. Hence, this study is of interest to people with RA and to Health care professionals involved in its management.

Abstract

last sentence – RA can be a motivator – revise to better reflect the results eg having RA can be a motivator pg 5 line 55/56

Strengths and limitations

Pg 5 line 61 – given than RA affects 3 times more men than women it would be expected that a study will have more women than men unless it was directly targeting men with RA. As this does not appear to be the case in this study this limitation should be removed or revised to say that views of men may be under represented and more research targeting men with RA is needed. As this was a qualitative study the intent is not to generalise the results

Introduction

pg 6/lines 74-77 – clarify that these studies 13-15 are about exercise and PA and not specifically HIIT.

Some additional references on HIIT in RA should be incorporated to give a more complete review of effects of HIIT (not papers I have authored) eg

https://doi.org/10.1093/geroni/igab046.3249

Ten weeks of high-intensity interval walk training is associated with reduced disease activity and improved innate immune function in older adults with rheumatoid arthritis: a pilot study | Arthritis Research & Therapy | Full Text

Effect of high-intensity interval training in physiotherapy primary care for patients with inflammatory arthritis: the ExeHeart randomised controlled trial | RMD Open

Expand on the rationale for including maintenance in the study – eg what is already known on the topic of maintenance of exercise in RA and what are the gaps from patient perspective relating to HIIT

Methodology

A key point is that participants in study had stable disease and low to moderate disease activity hence the results reflect that disease profile and cant be assumed to be relevant to high disease activity and unstable drug regime – this needs to be considered when presenting the wide dd range as a study strength

Emphasis is put on the 4 different exercise groups – consider include a table to describe their profile compared to the whole group and the 4 exercise groups to help the reader see how valid this emphasis is

How soon after the main study had completed did this study start?

What was the approach taken to undertake the content analysis eg see Krippendorff, K. (2018). *Content analysis: An introduction to its methodology*. Philadelphia, PA: Sage Publications

Pg 10 – line 142/143 – the point on data saturation – data saturation is met when no new themes emerge – what was extensive interview material in this case that indicated data saturation? At what point was it determined? Eg during initial data analysis? Please provide more detail on data saturation in context of how it should be reached.

Pg 9 – its confusing as presented to describe that an interview guide with open ended questions was used and then so say that at the answers to two broad questions were analysed. Were only 2 questions asked in the interviews? If more than 2, why were they not included in the analysis?

Please include the interview guide in the supplementary data

Was any software used to support the data management and analysis?

How was trustworthiness determined?

Unconscious bias – given the backgrounds of the people involved in the interviews and analysis was any work done with a wider group to check interpretation to address potential unconscious bias through familiarity with the subject area?

The wide disease duration is presented as a strength of the study being from 1-25 years yet the median is 3 years suggesting a short duration was more the profile of the participants. Are the authors confident that this is a strength of the study?

Results:

It may just be a formatting issue with how the file is prepared but Tables of quotes should come after the initial description of the theme and categories. Its more typical to embed the quotes in the body of the results as it helps the reader to see how the theme developed and relevant quates rather than referring to the table. Why was the table format chosen over embedding quotes?

As presented it's confusing re use of aims and categories to describe the results. More typically the themes are presented with their related categories rather than listing the categories under the aims with no themes. As presented it reads like the approach is more verification of the intervention rather than an openness to what could emerge around the patient experience.

Discussion

It is valuable to explore the factors that could have led to maintenance of exercise in some patients however, this reads like a new study in that it refers to theories eg SDT –How was self-efficacy explored in this study? Without further information on what the interviews focused on the discussion is currently over reaching on its conclusions eg pg 25 lines 430 – "*it is likely that these patients already had some degree of self-efficacy"* – was their baseline levels measured? If not this statement cannot be made. What theories informed the study eg did self-determination theory inform the question selection or frame of reference to analyse the results?

Same with intrinsic motivation – how was it measured? It cannot be stated based on the results in this study that patients who did not continue exercising can be categorised as "amotivated".

**Reviewer**	**2**
**Name**	**Swärdh, Emma**
**Affiliation**	**Karolinska Institutet**
**Date**	**19-Sep-2025**
**COI**	**None**

Comments to the Authors

Thank you for the opportunity to review this interesting and well-written manuscript. The topic is important, as there are patients who wish to continue or even explore intensive exercise despite living with chronic illness, and there is still a relative lack of patient perspectives on this matter.

Overall, the manuscript is clear and engaging. However, I have some suggestions that I believe would strengthen it further:

1. Background

While the background section is concise, I would have appreciated the inclusion of at least a brief reference to the qualitative literature on exercise in rheumatoid arthritis (RA), more than just barriers and facilitators. There has been substantial research in this field, even if not specifically focusing on HIIT, and acknowledging this body of work would help position the study more firmly within the broader context.

2. Methodology

I found the methodological description rather clear, but I would like to better understand why the second research question reached a higher level of

interpretation and abstraction compared to the first in ordet to be able to judge trustworthiness Was the data material richer for question two, or how was this difference in analytical depth determined? This is not sufficiently explained in the methods section, nor reflected upon in the discussion, and addressing it would enhance transparency.

3. Results

The results are straightforward and easy to follow. However, the subheadings for the categories of the first research question do not fully align in terms of grammatical structure and style (e.g., -ing form versus third person). I would suggest harmonizing these for consistency. Furthermore, the categories appear to operate at a higher level of interpretation than the subcategories, which is not fully mirrored in the way they are labeled. Further, try not to use the same wording in categories as in subcategories. Revising the naming conventions to better reflect interpretative levels would improve clarity.

4. Discussion

The discussion would benefit from a stronger integration with the existing body of research on RA and qualitative studies of exercise. I was particularly surprised that the meta-synthesis by Swärdh et al. (2021) is not cited, as it seems highly relevant to your findings, but also relate patient perceptions to the behavior change wheel and COM-B. Also, the original article " A Necessary Investment in Future Health": Perceptions of Physical Activity Maintenance Among People With Rheumatoid Arthritis" by Swärdh et al (2020), describe a similar theme as the one you describe for your second question, which would be great to delve into. It would also be interesting to read a reflection on if the perception for HIIT might vary from perception on other types of physical activity previously published and whether or not SDT is a good framework to use for interpretation for any intensity and types of exercise. Additionally, I would encourage the authors to elaborate further on the issue of saturation: What type of saturation do you consider to have been reached?

In summary, I find the manuscript to be of good quality and to make a meaningful contribution to the field. I recommend minor revision to address the points outlined above.

Best of luck with the revision!

VERSION 1 - AUTHOR RESPONSE

	**Reviewer 1**	

Abstract	Last sentence – RA can be a motivator – revise to better reflect the results eg having RA can be a motivator pg 5 line 55/56	The change has been made according to the reviewer's comments and marked with yellow in the manuscript:<br>*Nevertheless, having RA can be a motivator when exercise is viewed as an important part of medical treatment.*<br>Pg 4 line 55.

Strengths and limitations	Pg 5 line 61 – given than RA affects 3 times more men than women it would be expected that a study will have more women than men unless it was directly targeting men with RA. As this does not appear to be the case in this study this limitation should be removed or revised to say that views of men may be under represented and more research targeting men with RA is needed. As this was a qualitative study the intent is not to generalise the results	We agree that more women than men are affected by RA. Moreover, we also agree that the intent was not to generalise our findings to the broader RA population. This part has been removed from the limitation section accordingly.
Introduction	Pg 6/lines 74-77 – clarify that these studies 13-15 are about exercise and PA and not specifically HIIT. Some additional references on HIIT in RA should be incorporated to give a more complete review of effects of HIIT (not papers I have authored) eg https://doi.org/10.1093/geroni/igab046.3249 Ten weeks of high-intensity interval walk training is associated with reduced disease activity and improved innate immune function in older adults with rheumatoid arthritis: a pilot study | Arthritis Research & Therapy | Full Text<br><br>Effect of high-intensity interval training in physiotherapy primary care for patients with inflammatory arthritis: the ExeHeart randomised controlled trial | RMD Open<br><br>Expand on the rationale for including maintenance in the study – eg what is already known on the topic of maintenance of exercise in RA and what are the gaps	Thank you for being so observant. It is true that the references about exercise and PA are not specifically for HIIT. This has now been clarified. Further, more randomised controlled studies of HIIT on an intensity level corresponding to the present intervention (90-95% of maxHR) including other inflammatory joint diseases has now

	from patient perspective relating to HIIT	been added.<br>We have also expanded the part of maintenance of exercise in RA to better understand the rationale for including maintenance to exercising in the present study.<br><br>Pg 4 line 76-77, pg 5, line 79-81, 85

Methodology	A key point is that participants in study had stable disease and low to moderate disease activity hence the results reflect that disease profile and cant be assumed to be relevant to high disease activity and unstable drug regime – this needs to be considered when presenting the wide dd range as a study strength	We do agree that the patients were well-controlled RA and had a low disease activity and that our results cannot be assumed to be transferable to patients with RA with a higher disease activity. This has now been added in the limitation section (pg 28 line 544-547) as well as a bullet point in the article summary (pg 4, line 63-64).
	Emphasis is put on the 4 different exercise groups – consider include a table to describe their profile compared to the whole group and the 4 exercise groups to help the reader see how valid this emphasis is	We thank the reviewer for this suggestion. However, we believe that a more precise description of the patients´ profile in the 4 separate exercise groups could increase the risk that the individuals could be identified. We believe that a strength of the study is that patients from 4 separate groups from both study centres were included in the interviews.
	How soon after the main study had completed did this study start?	The interviews were conducted 3-6 months after the supervised HIIT sessions ended. This is now included in the Data collection section. Please see pg 7 line 143.

	What was the approach taken to undertake the content analysis eg see Krippendorff, K. (2018). Content analysis: An introduction to its methodology. Philadelphia, PA: Sage Publications	In this study, we applied qualitative content analysis as our methodological approach. Such analysis can be either descriptive or involve a deeper interpretation of interview texts. Our aim was to conduct a deeper, interpretive analysis of the interview material.<br><br>Various terms and concepts are used within content analysis. One such term is *domain*, which refers to the area or scope of the analysis. In this study, there were two domains: firstly, how patients experienced the supervised group-based HIIT training, and secondly, how they managed the training independently after the intervention ended.

		The analysis can be conducted inductively or deductively. An inductive approach means that the analysis is guided by the data itself, while a deductive approach is based on an existing theory or model.<br>This study employed an inductive approach.<br>In inductive analysis, the text can be interpreted at different levels of depth and abstraction. Two key concepts in this context are *manifest* and *latent* content. Manifest content refers to what is explicitly stated in the text, such as enumerations or concrete statements. In contrast, latent content involves a deeper interpretation of the underlying meaning. In this study a latent analysis was conducted.<br><br>It is essential that the researcher remains aware of issues related to credibility and quality throughout the entire research process and transparently

		reports the steps of the analysis.
	Pg 10 – line 142/143 – the point on data saturation – data saturation is met when no new themes emerge – what was extensive interview material in this case that indicated data saturation? At what point was it determined? Eg during initial data analysis? Please provide more detail on data saturation in context of how it should be reached	Thank you for highlighting the importance of describing saturation. We have clarified the part of saturation in the method section. Please see Pg 7-8 line147-149 *Inclusion was continued until data saturation was reached, whereby no new information addressing the research questions was given, and existing data was being replicated.*
	Pg 9 – its confusing as presented to describe that an interview guide with open ended questions was used and then so say that at the answers to two broad questions were analysed. Were only 2 questions asked in the interviews? If more than 2, why were they not included in the analysis?	The analysis is based on two broad questions. However, we realise that this part was unclear and have now clarified this in the Data analysis section Please see page pg 8 line 161-163. *The first question resulted in four categories and ten subcategories. In contrast, the second question generated richer material allowing a deeper, more latent and interpretative analysis that resulted in the development of two themes and their related subthemes.*
	Please include the interview guide in the supplementary data	Thank you for the recommendation. The interview guide is now included as a supplementary file accordingly. Supplement B. pg 7, line 142.
	Was any software used to support the data management and analysis?	No software was used.
	How was trustworthiness determined?	Thank you for your question. Trustworthiness was ensured through the whole process, from the planning of the study to the analysis and implications. To strengthen the trustworthiness of the analysing process, the two authors engaged in an open and critical dialogue, discussing all steps of the analysis until consensus was reached. This is described in the data

		analysis pg 8 line 163-165 and in strength and limitations section page 27, line 533-536.
	Unconscious bias – given the backgrounds of the people involved in the interviews and analysis was any work done with a wider group to check interpretation to address potential unconscious bias through familiarity with the subject area?	Thank you for the remark. Unconscious bias can arise in many areas of qualitative research. In the present study, the interviewer and data analysts were not involved in the intervention and had no personal or professional benefit from positive outcomes of the HIIT program. Furthermore, the interviewer had limited prior knowledge of treatment approaches for patients with RA, reducing the likelihood of bias toward any specific treatment. Importantly, the interviewer had no influence on the delivery of the intervention itself. We therefore believe that the risk of unconscious bias has been minimized in this study.
	The wide disease duration is presented as a strength of the study being from 1-25 years yet the median is 3 years suggesting a short duration was more the profile of the participants. Are the authors confident that this is a strength of the study?	It is true that the median disease duration of the participants in this qualitative study was 3 range 1-25 years. We do believe that it is a strength that not all patients had a shorter disease duration. However, we have removed disease duration from bullet-points.
Results	It may just be a formatting issue with how the file is prepared but Tables of quotes should come after the initial description of the theme and categories. Its more typical to embed the quotes in the body of the results as it helps the reader to see how the theme developed and relevant quates rather than referring to the table. Why was the table format chosen over embedding quotes?	Thank you for the suggestion to embed the quotes in the text. The quotes have now been embedded in the body of the result section accordingly.

	As presented it's confusing re use of aims and categories to describe the results. More typically the themes are presented with their related categories rather than listing the categories under the aims with no themes. As presented it reads like the approach is more verification of the intervention rather than an openness to what could emerge around the patient experience.	Thank you for your comment<br>In general, the analysis in a qualitative study may result in categories or themes. The categories should be mutually exclusive. Themes can include multiple categories or subthemes and serve to connect the underlying or latent content found within these categories or subthemes.<br><br>In the present study, the purpose of the study remained constantly present throughout the data analysis. But, for clarification, the aim has now been deleted from the result section.

		The two questions, or domains, were analysed separately. We have now clarified this part in the data analysis section. see page 8 line 161-163: *The first question resulted in four categories and ten subcategories. In contrast, the second question generated richer material allowing a deeper, more latent and interpretative analysis that resulted in the development of two themes and their related subthemes.* The methodological considerations have been elaborated, and changes are made in the strength and limitations section as well pg 28 line 549-553.

Discussion	It is valuable to explore the factors that could have led to maintenance of exercise in some patients however, this reads like a new study in that it refers to theories eg SDT –How was self-efficacy explored in this study? Without further information on what the interviews focused on the discussion is currently over reaching on its conclusions eg pg 25 lines 430 – "it is likely that these patients already had some degree of self-efficacy" – was their baseline levels measured? If not this statement cannot be made. What theories informed the study eg did self-determination theory inform the question selection or frame of reference to analyse the results?	Thank you for valuable comments on the discussion section. We have now revised this part of the discussion to better clarify how our findings are interpreted through the lens of SDT.<br>We have revised the text and made several changes in the discussion regarding the SDT according to your suggestions marked in yellow pg 25 line 468-469, 474-490
	Same with intrinsic motivation – how was it measured? It cannot be stated based on the results in this study that patients who did not continue exercising can be categorised as "amotivated"	We realize that the part of SDT was unclear. We have now revised this part of the discussion to better clarify how our findings are interpreted through the lens of SDT. For clarification, intrinsic motivation or amotivation were not measured. Accordingly, we have now clarified the part of intrinsic motivation and amotivation pg 26 line 500-502

	**Reviewer 2**	
Background	While the background section is concise, I would have appreciated the inclusion of at least a brief reference to the qualitative literature on exercise in rheumatoid arthritis (RA), more than just barriers and facilitators. There has been substantial research in this field, even if not specifically focusing on HIIT, and acknowledging this body of work would help position the study more	Thank you for suggestion to improve the introduction section. This part has now been extended to include also qualitative literatures to better reflect the rational of exercise in RA. Pg 4, line 76, pg 5, 79-81, 85.

	firmly within the broader context.	
Methodology	I found the methodological description rather clear, but I would like to better understand why the second research question reached a higher level of interpretation and abstraction compared to the first in ordet to be able to judge trustworthiness Was the data material richer for question two, or how was this difference in analytical depth determined? This is not sufficiently explained in the methods section, nor reflected upon in the discussion, and addressing it would enhance transparency.	Thank you for your comment. We agree that this part needs to be clarified and have now elaborated this in the data analysis section. Please see page 8 line 161- 163<br>The first question resulted in four categories and ten subcategories. In contrast, the second question generated richer material allowing a deeper, more latent and interpretative analysis that resulted in the development of two themes and their related subthemes.<br><br>Moreover, we have also made changes in the strength and limitations section in the discussion regarding saturation pg 28 line 549-553.

Results	The results are straightforward and easy to follow. However, the subheadings for the categories of the first research question do not fully align in terms of grammatical structure and style (e.g., -ing form versus third person). I would suggest harmonizing these for consistency. Furthermore, the categories appear to operate at a higher level of interpretation than the subcategories, which is not fully mirrored in the way they are labeled. Further, try not to use the same wording in categories as in subcategories. Revising the naming conventions to better reflect interpretative levels would improve clarity.	Thank you for your comment on the result section, and for your helpful suggestions regarding the headings of the subcategories. For harmonization, two categories have been renamed accordingly: *Belonging to a group facilitates exercise* and *Feeling confident when exercising*<br><br>The changes are made in table 2 and in the result section pg 13, line 228 and pg 14 line 252.

Discussion	The discussion would benefit from a stronger integration with the existing body of research on RA and qualitative studies of exercise. I was particularly surprised that the meta-synthesis by Swärdh et al. (2021) is not cited, as it seems highly relevant to your findings, but also relate patient perceptions to the behavior change wheel and COM-B. Also, the original article "A Necessary Investment in Future Health": Perceptions of Physical Activity Maintenance Among People With Rheumatoid Arthritis" by Swärdh et al (2020), describe a similar theme as the one you describe for your second question, which would be great to delve into. It would also be interesting to read a reflection on if the perception for HIIT might vary from perception on other types of physical activity previously published and whether or not SDT is a good framework to use for interpretation for any intensity and types of exercise.	Thank you for your reflections on the discussion. There are indeed many interesting aspects to explore further. As you mention, the body of research on RA and physical activity is quite extensive. However, the amount of research specifically addressing RA and high-intensity interval training (HIIT) remains limited. Therefore, we chose to focus our discussion on HIIT rather than physical activity in general, as we believe this better reflects the scope and aim of our study.<br><br>Thank you for the suggestion to discuss COM-B in comparison with SDT. We chose to use Self-Determination Theory (SDT) to discuss our findings, as it aligns well with the study's focus on motivation. This theoretical framework offered a meaningful lens through which to interpret participants' experiences and motivational processes related to exercise. While
		we acknowledge the relevance of the Behaviour Change Wheel and the COM-B model in broader behavioural research, we believe these frameworks encompass a wider scope that does not fully align with our findings. Moreover, including COM-B in the discussion might risk confusing the reader, which is why we chose to focus solely on SDT. However, we acknowledge the importance of the meta-analysis by Swärdh et al and this reference has now been added.

	Additionally, I would encourage the authors to elaborate further on the issue of saturation: What type of saturation do you consider to have been reached?	Thank you for highlighting the importance of describing saturation. In this study we have used "Data saturation".<br>Changes have been made in the in the method section Pg 7-8 line 147-149.<br>We have clarified the text as follows:<br>Inclusion was continued until data saturation was reached, whereby no new information addressing the research questions was given, and existing data was being replicated.

VERSION 2 - REVIEW

**Reviewer**	**2**
**Name**	**Swärdh, Emma**
**Affiliation**	**Karolinska Institutet**
**Date**	**01-Dec-2025**
**COI**	

Thank you for your thoughtful and thorough revisions. The revised manuscript addresses the previous concerns in a satisfactory manner. I believe it is now suitable for publication, and I wish you the best of luck with the remaining steps of the publication process.
